# Bioconjugation Strategy for Ceramic Membranes Decorated with Candida Antarctica Lipase B—Impact of Immobilization Process on Material Features

**DOI:** 10.3390/ma15020671

**Published:** 2022-01-17

**Authors:** Joanna Kujawa, Marta Głodek, Izabela Koter, Guoqiang Li, Katarzyna Knozowska, Wojciech Kujawski

**Affiliations:** Faculty of Chemistry, Nicolaus Copernicus University in Toruń, 7 Gagarina Street, 87-100 Toruń, Poland

**Keywords:** surface modification, organic spacer, enzyme immobilization, *Candida antarctica* lipase B, ceramic membranes, aluminum oxide

## Abstract

A strategy for the bioconjugation of the enzyme *Candida antarctica* lipase B onto titania ceramic membranes with varied pore sizes (15, 50, 150, and 300 kDa) was successfully performed. The relationship between the membrane morphology, i.e.,the pore size of the ceramic support, and bioconjugation performance was considered. Owing to the dimension of the enzyme (~33 kDa), the morphology of the ceramics allowed (50, 150, and 300 kDa) or did not allow (15 kDa) the entrance of the enzyme molecules into the porous structure. Such a strategy made it possible to better understand the changes in the material (morphology) and physicochemical features (wettability, adhesiveness, and surface charge) of the samples, which were systematically examined. The silane functionalization and enzyme immobilization were accomplished via the covalent route. The samples were characterized after each stage of the modification, which was very informative from the material point of view. As a consequence of the modification, significant changes in the contact angle, roughness, adhesion, and zeta potential were observed. For instance, for the 50 kDa membrane, the contact angle increased from 29.1 ± 1.5° for the pristine sample to 72.3 ± 1.5° after silane attachment; subsequently, it was reduced to 57.2 ± 1.5° after the enzyme immobilization. Finally, the contact angle of the bioconjugated membrane used in the enzymatic process rose to 92.9 ± 1.5°. By roughness (S_q_) controlling, the following amendments were noticed: for the pristine 50 kDa membrane, S_q_ = 1.87 ± 0.21 µm; after silanization, S_q_ = 2.33 ± 0.30 µm; after enzyme immobilization, S_q_ = 2.74 ± 0.26 µm; and eventually, after the enzymatic process, S_q_ = 2.37 ± 0.27 µm. The adhesion work of the 50 kDa samples was equal to 136.41 ± 2.20 mN m^−1^ (pristine membrane), 94.93 ± 2.00 mN m^−1^ (with silane), 112.24 ± 1.90 mN m^−1^ (with silane and enzyme), and finally, 69.12 ± 1.40 mN m^−1^ (after the enzymatic process). The materials and physicochemical features changed substantially, particularly after the application of the membrane in the enzymatic process. Moreover, the impact of ceramic material morphology on the zeta potential value is here presented for the first time. With an increase in the ceramic support cut-off, the amount of immobilized lipase rose, but the specific productivity was higher for membranes possessing smaller pores, owing to the higher grafting density. For the enzymatic process, two modes of accomplishment were selected, i.e., stirred-tank and cross-flow. The latter method was characterized by a much higher effectiveness, with a resulting productivity equal to 99.7 and 60.3 µmol h^−1^ for the 300 and 15 kD membranes, respectively.

## 1. Introduction

Enzymes have been widely used in biocatalysis, the degradation of pollutants, and wastewater treatment processes due to their high catalytic efficiency, high specificity, diverse range of catalytic reactions, and friendly reaction conditions, in order to develop green industrial processes and protect the environment [1,2,3,4,5]. For example, lipase can be used in alcoholysis, esterification, acidolysis, and transesterification in industrial processes [4]. To achieve the various applications of enzymes in industrial processes, the immobilization of enzymes on solid supports is frequently required [3]. Immobilized enzymes have demonstrated high chemical and thermal stability and high catalytic activity under practical conditions [4]. Moreover, the immobilization of enzymes on solid supports allows the recovery and reusability of enzymes, which significantly reduces the operational costs of enzyme catalytic processes [6].

The suitable selection of support materials is crucial to the efficiency of the enzyme immobilization process and the catalytic performance of immobilized enzymes. A variety of materials, e.g., ceramic nanoparticles [7,8,9,10,11], magnetic particles [12,13], polymeric and ceramic membranes [14,15,16], and metal–organic frameworks (MOFs) [17,18,19], have been utilized as supports for enzyme immobilization. Among these support materials, ceramic membranes are outstanding supports for enzyme immobilization in large-scale catalytic production processes, owing to their numerous advantages. The surface of ceramic membranes can be tuned/activated via chemical or physical processes to obtain hydroxyl groups allowing the attachment of linker molecules [20]. The pore structure and surface area of ceramic membranes can be tailored to improve the immobilization capacity. The utilization of membrane reactors can combine the catalytic reaction process with the simultaneous separation process, which increases the production efficiency and decreases the costs for product purification [16,20].

To achieve the efficient immobilization of enzymes on ceramic membranes, the membrane surface needs to be modified by linker molecules [15,16,21,22,23]. Hoog Antink et al. [16] modified yttria-stabilized zirconia (YSZ) capillary membranes with 3-(triethoxysilyl)propylsuccinic anhydride (TESPSA) or (3-aminopropyl) triethoxysilane (APTES) to investigate the impact of membrane surface modification on the enzyme (protease subtilisin A) attachment and the catalytic performance in the protein hydrolysis process. It was found that salinization did not influence the microstructure (porous properties and surface area) and mechanical strength of ceramic membranes. Modified carboxylated and aminated supports allow the conjugation of covalent enzymes onto the surface of a membrane by carbodiimide binding. The carboxylated ceramic support exhibited the least enzyme leaching, while the aminated ceramic support showed larger specific activity. The type of linker (silane) was of great importance for the initial adsorptive enzyme binding, the formation of covalent bonds, and the specific enzyme activity [16]. Zeuner et al. [21] fabricated YSZ ceramic membranes on a stainless steel metal support layer for the immobilization of alcohol dehydrogenase (ADH). In their work, ADH was immobilized on ceramic membranes by using two techniques, polyethyleneimine (PEI) coating, or APTES grafting, followed by glutaraldehyde (GA) activation and the consequent covalent immobilization of enzymes. It was found that the attachment via the covalent route substantially enhanced the activity, recyclability, and loading capacity of enzymes. In comparison to the APTES–GA technique, the PEI–GA technique enabled higher enzyme loading on the ceramic membranes due to the PEI network, which possessed a higher density of surface functional groups, providing more anchor points for GA and enzyme linkage [21]. The linker molecules play a crucial role in the generation of covalent bonds and the improvement of the immobilization efficiency and catalytic performance of enzymes. Therefore, the synthesis and selection of novel linker molecules can be crucial in the enzyme immobilization process.

Membranes containing immobilized enzymes have been intensively used in wastewater treatment processes, such as the selective removal of pesticides from vegetative water [24,25]; the removal of dyes [12,26]; the degradation of bisphenol A [2,27]; the degradation of antibiotics [22,28]; and the catalytic production of valuable chemicals, such as oligodextran production [29], the production of peptides from milk protein [30], the production of oligosaccharides [31,32], and the conversion of carbon dioxide into methanol [21]. De Cazes et al. [22] immobilized laccase on ceramic membranes by coating the ceramic support with a gelatin layer, followed by the GA activation and the subsequent enzyme attachment. The immobilized laccase exhibited the high degradation rate of 175 mg h^−1^ m^−2^ during the catalytic reaction test in solutions with 20 ppm of tetracycline. By using the same immobilization method, esterase was attached to TiO_2_ ceramic membranes for the degradation of erythromycin. Under the optimal conditions, e.g., pH at 4 and temperature at 45 °C, the immobilized esterase displayed after 100 h a degradation rate of 15.8 mg h^−1^. It was stated that the immobilized enzyme showed better stability in comparison to the free enzyme [28]. Zhang et al. [26] immobilized laccase on porous zeolite-like geopolymer membranes for the removal of crystal violet (CV) from wastewater. It was found that more than 99% of the CV was removed from a solution with a dye concentration of 5 mg/L by using the prepared composite membrane in a batch mode. In addition, the prepared composite membrane showed a removal efficiency of 90% for the dye over 6 h of filtration, indicating the high durability of the mmobilized enzyme. Zeuner et al. [23] attached ADH onto silicon carbide (SiC) membranes with a microporous structure by using the APTES–GA technique and the PEI–GA technique for the production of methanol from CHOH. It was pointed out that the ADH immobilized via the covalent pathway exhibited 2.5 times higher CHOH conversion and improved relative activity retention when compared with the physical method of ADH attachment.

Based on the above literary survey, it was possible to find research gaps and define the aims of this research. Specifically, there was no single work in which extended material studies had been completed to follow changes in physiochemistry, i.e., surface charge, morphology, and wettability, and to refer them to changes in enzyme reactivity. Such fundamental research, from the point of view of supports (i.e., ceramic porous membranes), is essential to better understand the functionalization and enzyme immobilization processes. The main goal of the present work was to find the common points between membrane morphology, particularly the pore size of ceramic supports, and bioconjugation performance. Furthermore, it was important to monitor how the physiochemical and material properties of the ceramics were adjusted after each step of modification, i.e., functionalization with the synthesized silane-based modifier and enzyme immobilization. Taking into account bioconjugation, the impact of enzymatic processes on material features was also evaluated.

## 2. Materials and Methods

### 2.1. Materials

Ceramic membranes with planar geometry and a dimension of 47mm with various molecular weight cut-offs (MWCO), i.e., 15, 50, 150, and 300 kDa, were bought from TAMI Industry (Nyons, France). The membranes possessed a multilayer structure, with the selective layer made of ZrO_2_ and the support layer made of TiO_2_. Ceramic powder of aluminum oxide (Al_2_O_3_) was acquired from Sigma-Aldrich (Warsaw, Poland). 1,6-diaminohexane (98+%) was purchased from Alfa Aesar (Kandel Germany). 3-isocyanatopropyltriethoxysilane (95%) and anhydrous dichloromethane were bought from abcr GmbH (Karlsruhe, Germany).

Vinyl acetate (≥99%) and glutaraldehyde (25% solution in water) and a Bio-Rad Protein Assay kit with a BSA standard were purchased from Sigma-Aldrich (Warsaw, Poland). (*R*,*S*)-1-phenylethanol was delivered by Fluka Chemie AG (Buchs, Switzerland). Lipase from *Candida antarctica* type B (Lipozyme CALB L) was provided by Novozymes (Bagsværd, Denmark). All reagents were used as received without any additional purification.

### 2.2. Analytical Methods—Ceramic Material Characterization

Attenuated total reflection–Fourier transform infrared spectroscopy (ATR–FTIR) was used for the assessment of ceramic material modification efficiency by applying Bruker Vertex 80v. The selected resolution was equal to 4 cm^−1^, and 512 scans were collected for each sample.

Thermogravimetric analysis (TGA) and differential thermal analysis (DTA) were performed at the temperature range of 25–1000 °C, with a heating rate of 20 °C/min under an ambient atmosphere of nitrogen. A Jupiter STA 449 F5 (Netzsch, Germany) device was used. The thermal resistance and sample stability were defined by applying TGA and DTG techniques. TGA was additionally selected for the purpose of functionalization efficiency assessment. The established method was followed, as described elsewhere [33,34].

High-resolution transmission electron microscopy (HR–TEM) was applied (Tecnai G2 F20 X-Twin, FEI Europe) to characterize carbon additives. The accelerating voltage was equal to 200 kV. Bright field imaging was carried out by TEM.

Surface charge was monitored via changes to the ζ potential in the pH range from 3 to 10.The ζ potential was tested according to potential streaming analysis using the SurPass3 Analyser (Anton Paar, Graz, Austria). The sample was fixed in the holder (clamping cell), allowing analysis of the entire sample without crushing or cutting. Such a procedure allowed us to analyze the surface charge after each step of the membrane modification. In the course of the analysis, laminar flow of electrolyte (1 mM KCl) was ensured. The required pH solution was adjusted by automatic dosing of 0.1 M HCl or NaOH. The pH range from 3 to 10 was used. The ζ potential calculation was performed based on the Helmholtz–Smoluchowski approach.

The particle size and zeta potential or ceramic metal oxide powders were measured with a Litesizer 500 (Anton Paar, Austria). The dispersion was prepared with deionized water (18 MΩcm) at a concentration of 1.5 mg/mL. Prior to the analysis, particles were dispersed with an ultrasonication bath for 5 minutes and subsequently diluted to the final concentration of 100 μg/mL. DLS (dynamic light scattering) and zeta potential by ELS (electrophoretic light scattering) were measured at 25 °C in deionized water (pH 5.8). The general-purpose approach was used, owing to samples’ low conductivity. Smoluchowski estimate was applied to translate the electrophoretic mobility into zeta potential [35].

Goniometric measurements coupled with a topography study were performed. A goniometer equipped with a 3D topography module was used (Attention Theta from Biolin Scientific, Gothenburg, Sweden). This method allowed us to determine apparent contact angle (CA) and contact angle corrected by roughness (CA_cor_). The topography profile was calculated by the application of the fringe projection phase-shifting method, working with the 3D topography module. Afterward, the scanned area of the sample was directly analyzed via the goniometric technique, i.e., sessile contact angle. One attention software was used for data acquisition. The goniometric measurements were carried out with DI water (18 MΩ cm) as a testing liquid. Equilibration time was equal to 5 s. CA was determined with ±0.5° accuracy at room temperature. The roughness was expressed by the S_q_ root mean square (RMS) determined according to ISO 25178 standard. Based on the contact angle values, work of adhesion (W_adh_) and spreading pressure (S) parameters were also determined [36,37,38,39].

### 2.3. Procedure for Grafting of Aluminum Oxide 

The aluminum oxide (Al_2_O_3_) was selected due to the availability of a much higher level of hydroxyl groups taking part in the grafting process. To describe the model reaction in detail, the modification was accomplished with aluminum rather than zirconium oxide. ZrO_2_ possessed more than 10 times fewer available reactive groups than Al_2_O_3_ [34].

The aluminum oxide powder was dried at 52 °C for 6 h. The modifier **1** was generated in situ and used without isolation for the grafting process. 1,6-hexanediamine (1.39 g, 12 mmol, 4 eq. (equivalent)) was dissolved in 30 ml of anhydrous dichloromethane, and the solution was cooled to −20 °C under nitrogen atmosphere. Next, the 3-isocyanatopropyltriethoxysilane (742 mg, 3 mmol, 1 eq.) was added dropwise. The ice bath was removed, and the mixture was stirred overnight at room temperature. The ratio of modifiers (in mmol) to the mass of metal oxide powder (in g) was expressed as the Q_MeOx_ parameter. Next, 15 mL of the DCM solution of modifier **1** was added to native Al_2_O_3_ (1.0 g). The suspension was shaken for 72 h at room temperature. After that, the grafted Al_2_O_3_ was centrifuged and washed with dichloromethane, aqueous HCl (pH 2–3), a solution of NaHCO_3_, and distilled water (five times in portions of 10 mL). The grafted powder of Al_2_O_3_ was dried over 24 h at 52 °C [40].

### 2.4. Functionalization Process of Ceramic Supports 

Membranes with different molecular cut-offs (15 kDa, 50 kDa, 150 kDa, and 300 kDa) were modified by analogy with the grafting process of the powder. Modifier **1** was obtained in the reaction of 3-isocyanatopropyltriethoxysilane and 1,6-diaminohexane in anhydrous DCM and subsequently applied during the functionalization process, without isolation in their pure forms as in Section 2.3 (Figure S1).

### 2.5. Enzyme Immobilization 

Lipase was attached onto the functionalized support via glutaraldehyde (GA). The functionalized ceramic membrane was treated with glutaraldehyde (GA) solution (1% *v*/*v* in phosphate buffer with pH 7, 10 mM) for 2 h at room temperature. The membrane was rinsed several times with deionized water to remove the residual GA. Lipozyme (CALB L) solution in phosphate buffer with pH 7 (10 mM) was passed through the membrane for 10 h at 12 °C with an axial velocity of ca. 1·10^−4^ m s^−1^. The membrane was subsequently washed with DI water and stored at 4 °C until use. The efficiency of the lipase load on the support was analyzed by the Bradford assay using BSA as a standard. The amount of immobilized lipase was defined as protein quantity and was calculated by subtracting the amount of free lipase in the solution from the total amount of the lipase used for immobilization.

### 2.6. Transesterification in the Membrane Reactor

**Scheme 1:**
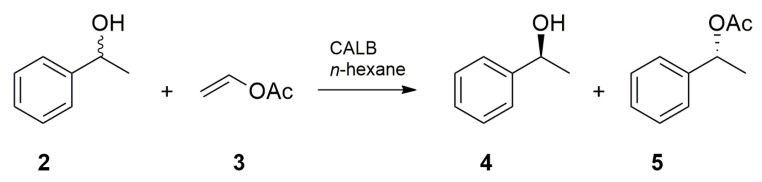
Scheme of the asymmetric transesterification of (*R*,*S*)-1-phenylethanol with vinyl acetate.

Activity and stereoselectivity of immobilized CALB were determined in a model transesterification of racemic alcohol **2** with vinyl acetate **3** as an acyl donor (Scheme 1). Transesterification of racemic alcohol **2** was carried out at 30 °C in an organic solvent system consisting of 25 mL of n-hexane as solvent dissolving 0.026 g (1 mmol) of (*R*,*S*)-1-phenylethanol **2** and 0.034 g (2 mmol) of vinyl acetate **3**. The process was carried out in a membrane reactor with constant stirring (150 rpm) under a magnetic stirrer (stirred-tank reactor) or with a reaction mixture circulating in the cross-flow membrane reactor system with an axial velocity of ca. 1·10^−5^ m s^−1^. Aliquots were withdrawn at specified time intervals from the reaction mixture and analyzed by chiral HPLC to evaluate the percentage of conversion and the enantiomeric excess of reactant and products. HPLC conditions: Daicel Chiralcel OD-H (250 mm × 4.6 mm, 5 µm) column with a Chiralcel OD precolumn; n-hexane/propan-2-ol 90/10 (*v*/*v*), 1 mL/min, Shimadzu SPD-10A VP UV-Vis detector (254 nm) (Shimadzu Europe, Duisburg, Germany).

## 3. Functionalization of Ceramic Supports

### 3.1. Functionalization of Ceramic Powder Al_2_O_3_

In the case of ceramic material functionalization, it is essential to comprehend the grafting yield and the impact of the overall process on various parameters, i.e., the material and physicochemical properties. To do so, the analysis began with the modification and characterization of the ceramic powders. This made it possible to analyze the materials with a wider range of instrumental techniques that would be impossible to implement if working with the membranes directly. To verify the functionalization efficacy, ATR–FTIR, TGA, HR–TEM, and the goniometric method were employed. Taking into account that the level of available hydroxyl groups was the decisive factor, aluminum oxide (Al_2_O_3_), which held the highest number of OH groups, was chosen in this part of the research.

**Figure 1:**
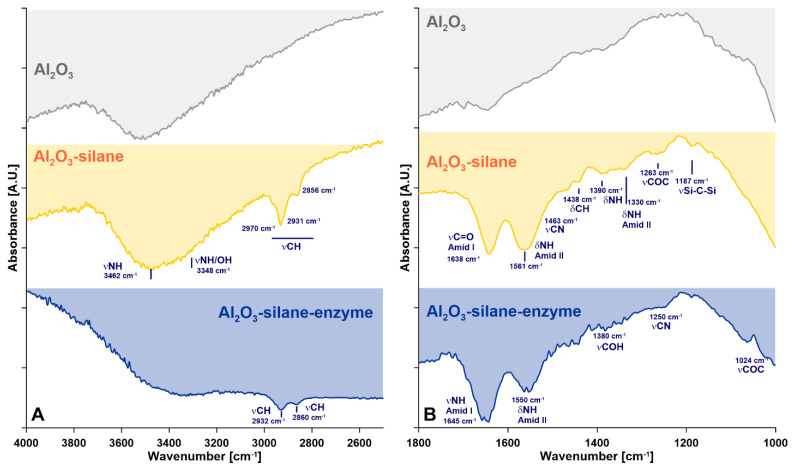
ATR–FTIR spectra of pristine Al_2_O_3_ and Al_2_O_3_ modified with silanes: (**A**) range of 4000–2500 cm^−1^, (**B**) range of 1800–1000 cm^−1^.

**Figure 2:**
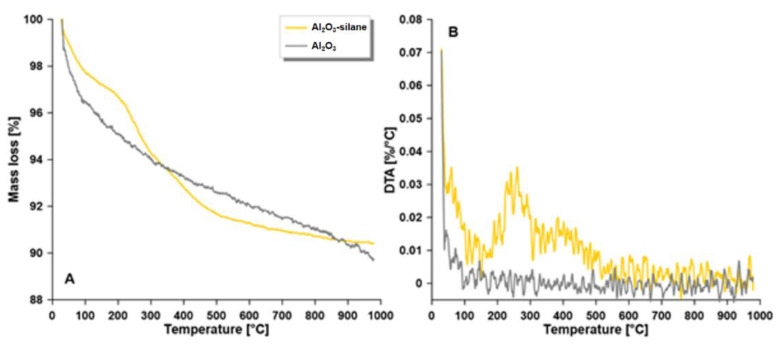
Thermal properties: TGA (**A**) and DTA (**B**) of the investigated samples.

The ATR–FTIR spectra were gathered and are presented in Figure 1 and Figure S1. It was proved that the modification process with the synthesized silane-based modifier (Figure S1) was successful and was accomplished via the chemical method. The characteristic bands of the grafting agent as well the enzyme were found in the dactyloscopic regions 1300–900 cm^−1^ (Figure 1 and Figure S1); 1190–1100 cm^−1^ (-Si-O-Si- stretching mode) (Figure 2); and 800–700 cm^−1^ (-Si-O-Si-) (Figure S2), and in a further range of the IR spectra. The asymmetric vibration bands of CH_3_ and the in-plane deformation of CH_2_ were observed at 1462 and 1438 cm^−1^. Moreover, at 1263 cm^−1^, bands coming from -C-O-C- bonding were detectable, and at 1130 cm^−1^ bands from -C-O- stretching associated with the alkyl chain of modifiers [3]. An important part was related to the characteristic bands of NH (γ_N-H_ 3348 cm^−1^; δ _N-H_ 1561 cm^−1^, Amide II; δ _N-H_ 1330 cm^−1^, Amide III), and C=O groups (γ_C=O_ 1638 cm^−1^, Amide I) were also found. It was important to note that no bands at 950–900 cm^−1^ came from -Si-OH (-Si-Oet-stretching mode), explaining the high level of ethoxy (-OEt) group utilization in the course of the grafting process [1,2,3]. For the Al_2_O_3_ modified with silanes, bands placed between 2850 and 2970 cm^−1^ showed asymmetric and symmetric CH_2-_stretching vibrations of the alkyl chain of the modifiers (Figure 1). However, broad bands visible at 3462 cm^−1^ and 3348 cm^−1^ proved the presence of available terminal amine groups that were crucial for the next step of the modification, i.e., enzyme attachment.

Additionally, on the spectrum of Al_2_O_3_, the characteristic bands from the enzyme, i.e., lipase, were visible. The observation of the vibrations of amide I, amide II, and amide III bonds in the structure of the enzyme at 1645, 1550, and 1250 cm^−1^ (Figure 1) was significant. The presence of these bands is said to prove efficient protein immobilization, as reported in the literature [4,5]. Furthermore, changes in the intensity of the signals generated by particular functional groups, i.e., hydroxyl and amine, indirectly proved the successful immobilization of the lipase on the ceramic surface.

To determine the thermal stability of the modified ceramic powder, thermal gravimetric analysis was performed (Figure 2A) with the monitoring of the decomposition temperature (T_d_). The T_d_ for the modified sample was equal to 260 °C, and the decomposition took place in the multistep process according to the derivative of the TGA plot (Figure 2B). TGA allowed us to not only determine the stability but also the effectiveness of the grafting process with the support of ATR–FTIR. The efficiency of the modification was established based on the number of hydroxyl groups available before and after modification (Equations (1) and (2)) [6,7]. The modification was characterized by a very high yield, equal to 96.2%.

(1) $$n_{OH}\left[mmol\cdot g^{-1}\right]=\frac{2\left[WL\left(T_{0}\right)-WL\left(T_{f}\right)\right]}{100\cdot M_{H2O}}$$

(2) $$n\left[OH\cdot m^{-2}\right]=\left(\frac{2\left[WL\left(T_{0}\right)-WL\left(T_{f}\right)\right]}{100\cdot M_{H2O}}\right)\left(\frac{N_{A}}{S_{BET}}\right)$$

where *WL*(*T*_0_) − *WL*(*T_f_*) stands for the mass loss (wt%) in the temperature range of interest (110–230 °C), *M_H2O_* is the water molar mass, *N_A_* is the Avogadro constant, and *S_BET_* is the specific surface area derived from BET.

**Figure 3:**
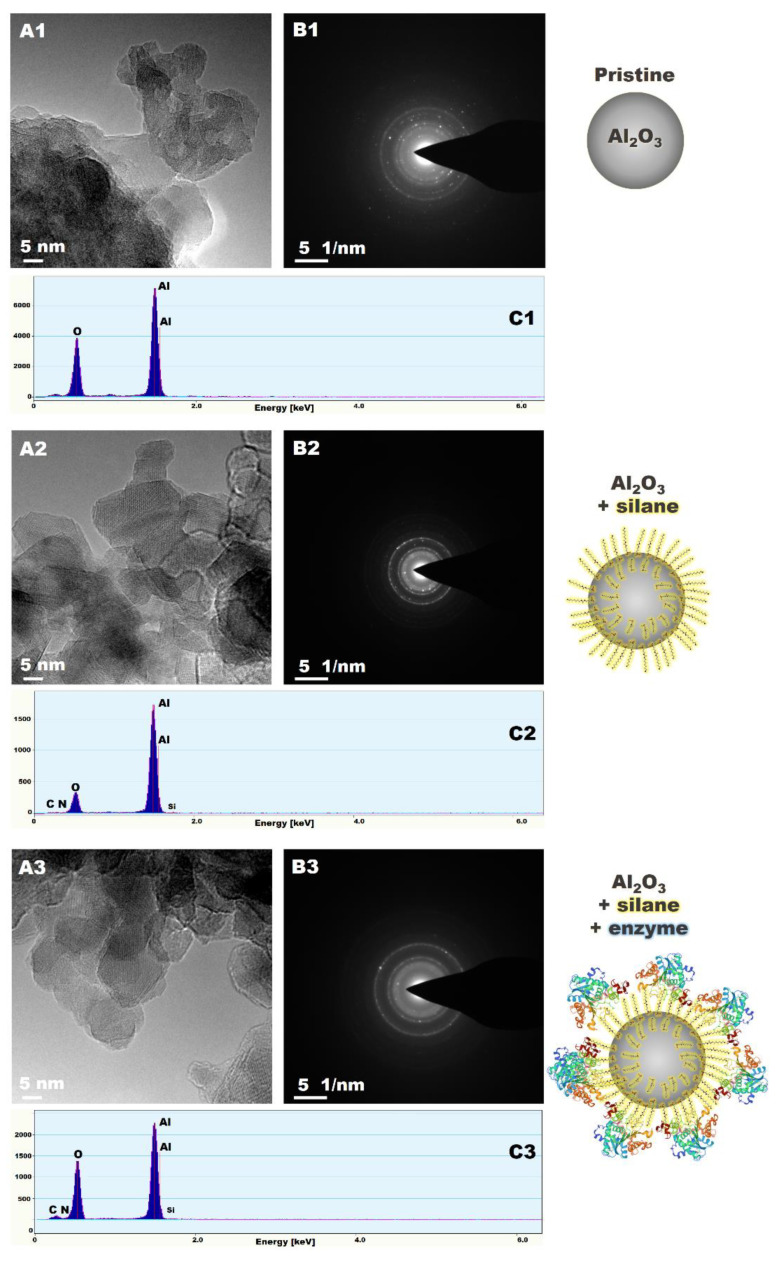
HR–TEM: bright field (**A1**–**A3**), SEAD images (**B1**–**B3**), and EDX spectra (**C1**–**C3**). **A1**,**B1**,**C1**: pristine Al_2_O_3_; **A2**,**B2**,**C2**: Al_2_O_3_ functionalized with silane; **A3**,**B3**,**C3**: Al_2_O_3_ functionalized with silane and enzymes.

According to the HR–TEM data, it was proven that the ceramic powder was homogenously modified (Figure 3). To verify the highly effective functionalization with the silane-based modifier, SEAD (selected electron area diffraction) and bright field imaging were carried out. Even though only slight differences in the transmission images were noticed, the alteration of the SEAD diffraction pattern was clearly visible (Figure 3). For the pristine Al_2_O_3_, the characteristic dots were visible, displaying the crystalline structure of aluminum oxide (Figure 3B1). For the material, after the silanization process, the characteristic rings coming from the amorphous phase were visible (Figure 3B2). Moreover, the material with the attached silanes and enzymes possessed practically only the rings in the SEAD image (Figure 3B3). It should be highlighted that the size of the rings increased when compared with the sample modified only with the silane-based modifiers. The rise in the rings’ size was evidence of attachment additionaly a bigger structure in comparison to the Al_2_O_3_ modified by silane alone. Taking into account the EDX analysis for the material grafted with silane, an increase in the carbon counts and the appearance of new peaks from silicone and nitrogen were found (Figure 3C2). Moreover, when the enzyme was immobilized on the surface of the grafted Al_2_O_3_, a significant increase in carbon and oxygen was observed (Figure 3C3).

**Figure 4:**
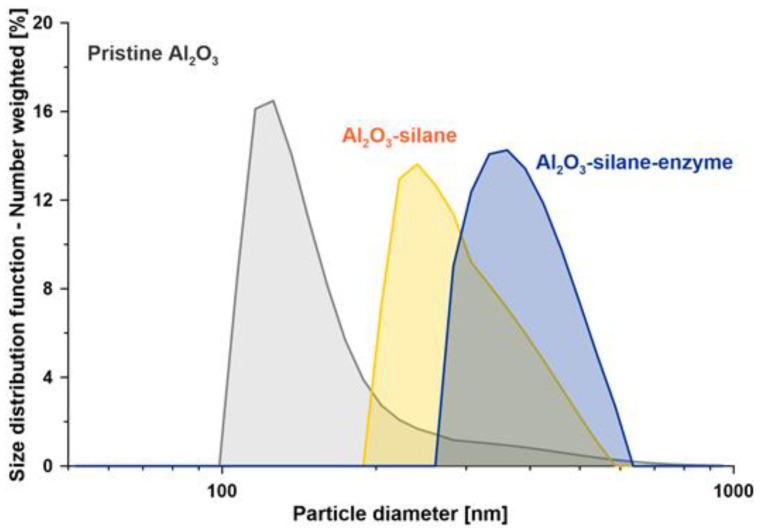
Changes in hydrodynamic diameter determined by DLS after silane functionalization and enzyme immobilization.

The diffraction laser scattering (DLS) and electrophoretic light scattering (ELS) measurements of the Al_2_O_3_ powder revealed that, as a consequence of the modification process, there was an increase in the hydrodynamic diameter from 125 ± 11 nm to 243 ± 15 nm. Further increment was found after attachment of the enzyme (Table 1, Figure 4). Due to the formation of a more branched structure in the course of the modification steps, a reduction in the electrophoretic mobility of ca. 20% was seen (Table 1). The tendency towards an agglomerate formation was expressed by the polydispersity index, which increased with the consecutive steps of the modification. For the pristine Al_2_O_3,_ the polydispersity index was equal to 20%, whereas after silanization and enzyme immobilization it rose to 24% and 45%, respectively. All the modified samples were characterized by very high stability, confirmation of which was found in the distribution of the hydrodynamic diameter in water with a monodispersed population (Figure 4). Based on the ELS carried out in ultrapure water (pH = 5.8), it was demonstrated that the surface charge was influenced by the modification. The fact that after the silane attachment, the amine terminal groups were easily available might have influenced the zeta potential. The value was slightly reduced from 42.9 ± 2.2 mV to 35.2 ± 1.8 mV when comparing the pristine sample with the sample modified with silane. Nevertheless, the enzyme attachment increased the zeta potential to 39.6 ± 1.3 mV. Furthermore, the decline in electrophoretic mobility should be linked to the high level of enzyme loading on the surface. After enzyme addition, the surface becomes less movable and uniform. The measured value of the pristine Al_2_O_3_ was in good agreement with the literature data [41]. It should be pointed out that zeta potential is subjected to particle size [42]. Particles in an aqueous solution, in the size range up to 1 μm, classically carry an electric charge and possess the tendency to approach one another due to the Brownian movement rule [43]. Based on the Derjaguin–Landau–Verwey–Overbeek (DLVO) concept [44], when the kinetic energy of the colliding particles is sufficient to overwhelm the energetic barrier from the electrostatic repulsive force, the van der Waals attractive force succeeds and lets the particles adhere strongly together. The presented model is valid for coagulation or flocculation, thus raising the size of colloidal aggregates [45]. In water, the stability of particles of submicron size, as well as nanoparticles, is related to electrostatic interactions or double-layer repulsive forces if only DLVO forces are present. It should be pointed out that electrostatic interactions or double-layer repulsive forces are much more intense than van der Waals attractive forces [46]. For the range of non-DLVO attractive forces, such as hydrophobic forces, connection by patch charging, adsorbed additives, and hydrogen bonding of the functional groups of adsorbed additives dominate [47]. The cause of the substantial difference between zeta potential for the pristine, functionalized, and immobilized materials was the addition of first the linkers and then the enzymes, as well as the moderation of the hydrophilicity/hydrophobicity features.

#### Ceramic Membrane Functionalization

**Figure 5:**
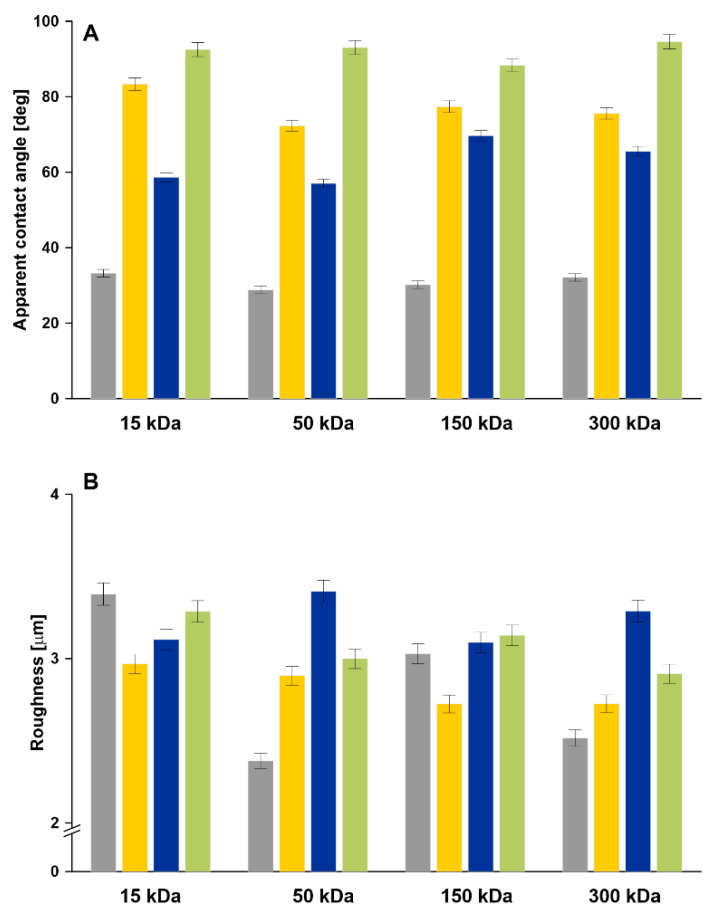
Differences in apparent contact angle (**A**) and roughness (**B**) of the investigated membranes: pristine samples, samples modified with silane, samples immobilized with the enzyme, and samples applied in the process.

**Figure 6:**
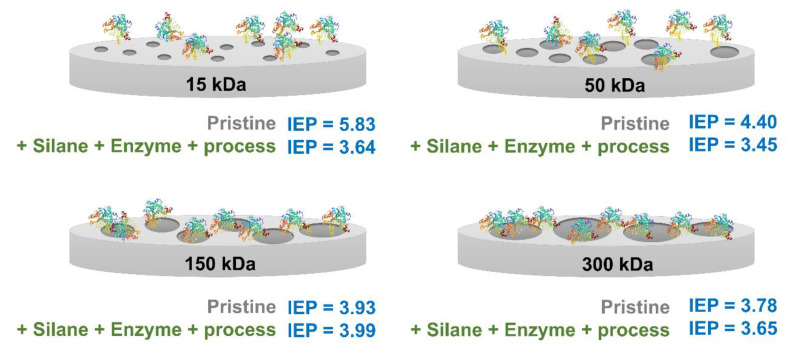
Relation between surface morphology (MWCOs) and possible location of grafting molecules and enzymes and the changes in isoelectric point (IEP) of the surface.

The membranes modified with both silanes and enzymes were assessed by the implementation of the goniometric method. The following parameters were determined: contact angle, apparent and corrected by the roughness; work of adhesion; and spreading pressure. It was essential to characterize these parameters to gain comprehensive information on how the designed treatment influenced the materials’ wettability and physiochemistry (Figure 5). During the study, the goniometric measurements were carried out for the pristine membrane, the sample modified with silane, and the immobilized enzyme. Moreover, the wettability study was carried out for the final hybrid material containing silane and the enzyme applied in the enzymatic process (Figure 5). It should be highlighted that during the research, the ceramic membranes with four different molecular weight cut-offs (15, 50, 150, and 300 kDa) were applied to evaluate the impact of the membrane morphology on the modification and finally on the enzymatic process effectiveness. Bearing in mind the significant influence of the material roughness and heterogeneity on the contact angle values, such behavior was noticeable even for the pristine membrane. Moreover, independently of the pore size of the ceramics, an increase in the contact angle was noticed that proved the success of the grafting process. An impact of the MWCO on the contact angle values was clearly visible (Figure 5 and Figure S3). The materials with the smallest MWCO, and consequently with smaller pore size, were characterized by a higher contact angle value. This can be explained by the fact that the membrane with the smallest pore size did not allow the entrance of the grafting silane molecules to the entire porous structure (Figure 6). As a consequence, more grafting molecules were available on the top of the ceramic membranes. The abovementioned observation is coherent with the roughness parameters (Figure 5). The reduction in the contact angle and increase in the roughness parameters after the immobilization of the enzyme is consistent with the literature data [5,48]. An interesting tendency was noticed for the membranes applied in the enzymatic process. For all the abovementioned samples, a significant increase in the contact angle, independently of the material morphology, was observed. On the other hand, there was no relation with roughness in the case of the membrane after the process (Figure 5). Considering both parameters together, i.e., the contact angle and the roughness, it was seen that the most significant changes were found for the membranes with bigger pores (Figure S3).

To comprehend the wettability feature more effectively, additional parameters were calculated, i.e., the work of adhesion (W_adh_), spreading pressure (S), and a contact angle corrected by the roughness (CA_cor_). The collected data are presented in Table 2. The corrected contact angle (CA_cor_) is a factor which takes into account the micro- and macroscale roughness. In the case of the pristine samples, the corrected CA values were slightly higher than the apparent CA values. Such behavior can be linked with the occurrence of a microdomain with hydrophobic characteristics on the hydrophilic surface. On the other hand, the modified materials with the contact angle close to 90° were characterized by slightly lower values of the corrected CA compared with the apparent CA values. The explanation for this should be sought in the likelihood of the presence of hydrophilic microareas (Table 2). The spreading pressure as well as the work of adhesion are subtle factors in the evaluation of the physiochemical features of modified materials.

The materials possessed a value of spreading pressure close to zero when they had developed a more hydrophilic character. Out of the collected data, the values closest to zero were those characterizing the pristine sample, which were in the range of −12.16 ± 0.28 mN m^−1^ to −9.19 ± 0.21 mN m^−1^ (Table 2). Moreover, slight differences among these values were noticed, which were related to the varied morphology. The observed results were coherent and additionally explained by the surface charge differentiations. As a consequence of the functionalization process with the silane-based modifiers, the spreading pressure values were diminished and were found to be in the range of −64.56 ± 0.35 mN m^−1^ to −50.67 ± 0.33 mN m^−1^. Furthermore, after enzyme immobilization to the surface by covalent bonding, the values of S rose and were placed closer to zero. Finally, after the application of the bioconjugate materials in the enzymatic process, a reduction in the spreading pressure was visible and ranged between −78.64 ± 0.41 mN m^−1^ and −70.77 ± 0.48 mN m^−1^.

Considering the work of adhesion, a reduction in the W_adh_ was observed for all of the modified samples when in their pristine conditions. Such changes are associated with the occurrence of weaker interactions between the water molecules and material, correlating directly with a higher hydrophobicity. Among the modified ceramic membranes, slightly higher values of W_adh_ were found after enzyme immobilization, guaranteeing the strongest interactions between the enzyme-rich surface and water molecules. Finally, the most reduced adhesion properties of the modified ceramics were seen for the materials after application in the enzymatic process [49,50].

**Figure 7:**
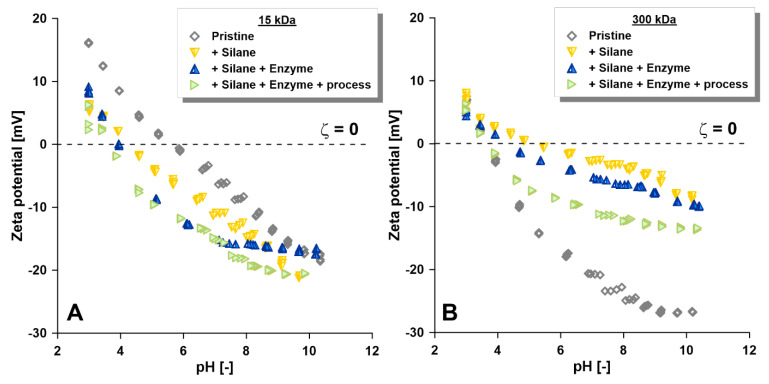
Zeta potential in the pH range of 3–10. Impact of all modification steps for the membranes with the smallest, 15 kDa (**A**), and the biggest, 300 kDa (**B**), pore size (MWCO).

**Figure 8:**
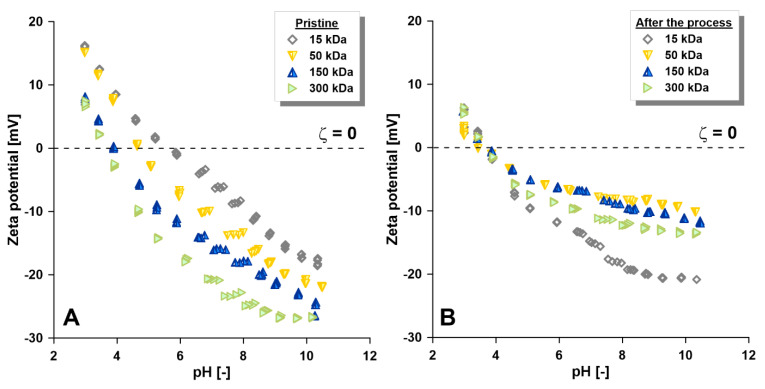
Zeta potential in the pH range of 3–10. Impact of membrane morphology on surface charge (**A**) and stability of the membrane after the enzymatic process (**B**).

To understand in detail the alterations to the hybrid materials’ features, zeta potential analyses were performed to study the affinity between the silane, enzyme, and ceramic support (Figure 6). The zeta potential (ζ) measurements with potential streaming analysis were informative and shed light on the subtle differentiation in the surface charge after the modifications (Figure 6, Figure 7 and Figure 8). First of all, the morphology of the ceramic support has a substantial influence on the surface charge (Figure 6 and Figure 7). The membranes with a smaller pore size (Figure 6 and Figure 8A) possessed less negative charge on their surfaces. More negative values were observed gradually as the pore size of the ceramics increased. Moreover, the isoelectric points (IEP) for the pristine supports were reduced as the pore size of the membrane was increased. The 15 kDa membrane was characterized by the IEP 5.85, and the membranes with bigger pores possessed the following values of IEP: 4.40 (50 kDa), 3.93 (150 kDa), and 3.78 (300 kDa) (Figure 6). In the case of the membranes modified with silanes, the surface charge was strongly influenced by the pore size. In the case of the 15 kDa membrane, a slight reduction in the zeta potential values was observed (Figure 7A). However, for the sample with the biggest pores, i.e., 300 kDa, an opposite tendency was noticed. In this particular case, the pristine material was characterized by a highly negative charge on the surface. Nevertheless, a significant enhancement in the positive charge as an effect of the silanization process was found. The most significant impact was seen in the base range of the pH (Figure 7B). On the other hand, independently in the membrane morphology after bioconjugation and the application of the membrane in the enzymatic process, an improvement in the negative character of the surfaces was noticed. Such behavior is typical for bioconjugates and is considered as a fingerprint of protein settlement, leading to a decrease in the overall electrostatic potential [51,52]. It should be pointed out that the membrane supports were selected in order to provide the opportunity to study the impact of membrane modification. For this reason, the material with the smallest pore size was chosen to ensure the impossibility of the entrance of the enzyme, with a pore size of ca. 20 kDa. Confirmation of the abovementioned behavior can be seen in the changes to the surface charge of the materials modified and applied in the enzymatic process (Figure 8B). The zeta potentials for the 15 kDa material were much smaller than for the other samples, where no significant differences were observable. This was related to the fact that for the 50 kDa, 150 kDa, and 300 kDa membranes, the enzyme and silane molecules could be located inside the pores, and their access to the surface was limited. 

### 3.2. Enzyme Immobilization

**Figure 9:**
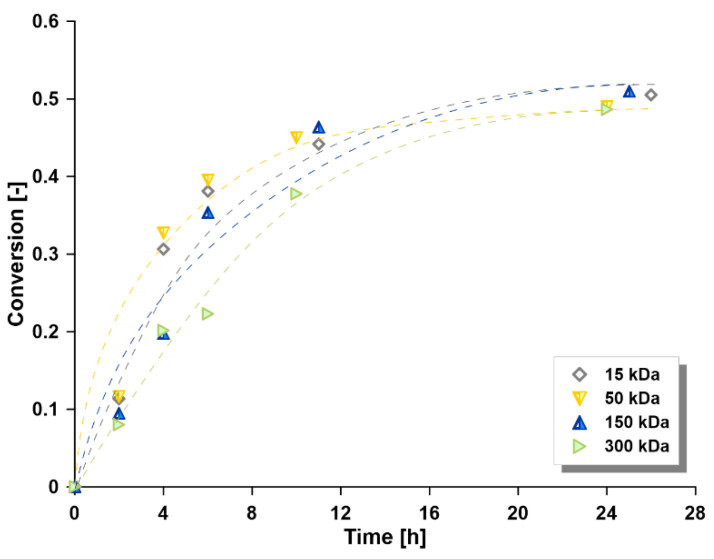
Comparison of conversion level for all of the investigated membranes.

**Figure 10:**
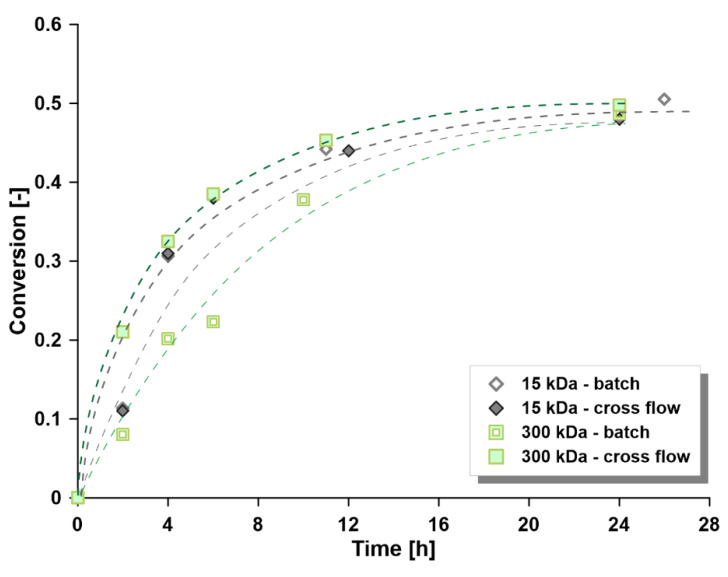
Conversion rate as a function of reaction time with CALB lipase immobilized in a 15 and 300 kDa cut-off membrane.

From the above results (Table 3, Figure 9 and Figure 10), one can see the clear dependence of the amount of lipase immobilized on the membrane pore size. The higher the cut-off, the higher the lipase content of the membrane. This is in accordance with the predictions. Lipase from *Candida antarctica* B (CALB) (EC 3.1.1.3) is composed of 317 amino acid molecules and has a molecular weight of 33,273 Da and an approximate size of 30 Å × 40 Å × 50 Å [53]. In the case of the densest membranes (15 kDa), lipase binds only on the surface, while in the case of membranes with a higher cut-off, it is more likely to enter the membrane pores and immobilize inside the active layer (Figure 6).

In contrast, the initial activity values, especially when converted to the amount of lipase, indicated that lipase was more productive in the membranes with a lower cut-off. Since the transesterification process was carried out in a membrane stirred-tank reactor, the reactants had to diffuse to the membrane pores and then diffuse outwards, which may have affected the lower specific activity of the lipase (Figure 9, Table 3).

To better examine this effect, analogous transesterification processes were performed in a cross-flow membrane reactor for the membranes of the extreme cut-offs: 300 and 15 kDa (Figure 9). The results for these processes compared to the stirred-tank system are shown in the Figure 10 and Table 4.

As can be seen, the process efficiency for the 300 kDa cut-off membrane was clearly higher for the flow reactor than for the stirred-tank reactor, while a similar effect was not observed for the 15 kDa cut-off membrane. A possible explanation for this is that the flow of the reaction mixture through the membrane reduced the diffusion restrictions. Since in the case of a membrane with a cut-off smaller than the size of the enzyme molecules, the biocatalyst is immobilized mainly on the surface, the catalytic activity of the lipase was not significantly affected by the process method. The slightly lower lipase activity in this membrane may have been due to the fact that the membrane was previously subjected to several zeta potential tests over a wide pH range (4–10), which may have influenced the slight decrease in the catalytic activity compared to the first process.

## 4. Conclusions

A strategy for the bioconjugation of the enzyme *Candida antarctica* lipase B onto titania ceramic membranes with a varied pore size (15, 50, 150, and 300 kDa) was successfully accomplished. The link between the membrane morphology, specifically, the pore size of the ceramic support, and the bioconjugation performance was presented. The selection of membranes with pore sizes smaller and larger than the enzyme dimensions gave an astonishing opportunity to study the impact of modification on the material as well as the physicochemical properties. During the study, the wettability, goniometry, surface charge, and adhesion features were systematically characterized. Finally, the comprehensively described membranes were used in the enzymatic process. It was essential and very informative to characterize the membranes after each modification step, achieving a comprehensive overview of the material features. To establish the effectiveness of the grafting process and the enzyme immobilization, the treatment was initially accomplished using ceramic powders. Afterward, the ceramic membranes were modified in an analogous way. The effectiveness of the modifications was very high, equal to 92%. The silane linker and the enzyme were both covalently attached to the ceramic support. By considering the collected data, it was possible to comprehend how both treatments changed the physicochemical features and to evaluate the influence of the supports’ morphology. Significant changes in the contact angle, roughness, and zeta potential after each step of functionalization were observed. Particularly after the application of the membrane in the enzymatic process, the material and physicochemical features changed substantially. The novel element of this study was to measure the contact angle corrected by roughness to acquire precise information about the changes in the wettability features. Furthermore, this is the first study in which the impact of ceramic material morphology on the zeta potential is presented. This influence was observed also during the enzymatic process. In the case of the 300 kDa membrane, a very high efficiency of the process, characterized by the productivity of 99.7 µmol h^−1^, was observed. The same process accomplished with the 15 kDa membrane possessed productivity on much lower level, equal to 60.3 µmol h^−1^. Moreover, the modes of the process were compared, and it was found that the cross-flow process was much more efficient than the stirred-tank process. The productivity for the cross-flow and stirred-tank methods were 99.7 µmol h^−1^ and 43.6 µmol h^−1^ for the 300 kDa sample, and 60.3 µmol h^−1^ and 68.9 µmol h^−1^ for the 15 kDa sample, respectively. The presented findings demonstrate that the pore size of the ceramic support plays a significant role in processes with chemically immobilized lipase. The physicochemical properties of the substrate depend on the cut-off, which influences the amount and availability of the enzyme. However, it is important to note that many other factors affect the efficiency of the biocatalyst, such as the type of enzyme, the mode of enzyme binding, the environment, or the membrane reactor configuration; therefore, the conclusions cannot be generalized.

## Figures and Tables

**Table 1:** DLS and ζ (zeta potential) characteristics of the ceramic materials.

Sample	Hydrodynamic Diameter [nm]	ζ Potential [mV]	Conductivity [µS cm^−1^]	Electrophoretic Mobility [µm cm V^−1^ s^−1^]
**Al_2_O_3_**	125 ± 11	42.9 ± 2.2	5.8 ± 0.7	3.35 ± 0.37
**Al_2_O_3_–silane**	243 ± 15	35.2 ± 1.8	3.6 ± 0.3	3.08 ± 0.25
**Al_2_O_3_–silane–enzyme**	357 ± 23	39.6 ± 1.3	2.4 ± 0.5	2.74 ± 0.21

**Table 2:** Wettability study of the investigated samples: contact angle corrected by the roughness (CA_cor_), work of adhesion (W_adh_), and spreading pressure (S).

Sample	CA_cor_ [deg]	W_adh_ [mN m^−1^]	S [mN m^−1^]
15 kDa			
Pristine 15 kDa	33.6 ± 1.5	133.4 ± 2.2	−12.16 ± 0.28
+ Silane	83.5 ± 1.5	81.0 ± 2.0	−64.56 ± 0.35
+ Silane + Enzyme	58.9 ± 1.5	110.4 ± 2.1	−35.20 ± 0.38
+ Silane + Enzyme + Process	92.3 ± 1.5	69.9 ± 1.5	−75.72 ± 0.32
50 kDa			
Pristine 50 kDa	29.1 ± 1.5	136.41 ± 2.2	−9.19 ± 0.21
+ Silane	72.3 ± 1.5	94.93 ± 2.0	−50.67 ± 0.33
+ Silane + Enzyme	57.2 ± 1.5	112.24 ± 1.9	−33.36 ± 0.30
+ Silane + Enzyme + Process	92.9 ± 1.5	69.12 ± 1.4	−76.48 ± 0.55
150 kDa			
Pristine 150 kDa	30.7 ± 1.5	135.40 ± 2.1	−10.20 ± 0.31
+ Silane	77.4 ± 1.5	88.68 ± 2.0	−56.92 ± 0.61
+ Silane + Enzyme	69.8 ± 1.5	97.94 ± 1.9	−47.66 ± 0.55
+ Silane + Enzyme + Process	88.4 ± 1.5	74.83 ± 1.8	−70.77 ± 0.48
300 kDa			
Pristine 300 kDa	32.5 ± 1.5	134.20 ± 2.1	−11.40 ± 0.25
+ Silane	75.6 ± 1.5	90.90 ± 2.0	−54.70 ± 0.36
+ Silane + Enzyme	65.7 ± 1.5	102.76 ± 1.9	−42.84 ± 0.32
+ Silane + Enzyme + Process	94.6 ± 1.5	66.96 ± 1.4	−78.64 ± 0.41

**Table 3:** Results of determination of lipase activity in enzyme membranes depending on membrane cut-off.

Cut-Off	Amount of Lipase Immobilized [mg]	Productivity [µmol h^−1^]	Productivity/mg of Lipase[µmol h^−1^mg^−1^]
15 kDa	0.84 ± 0.18	68.9 ± 14.5	82.1 ± 34.9
50 kDa	1.49 ± 0.18	75.0 ± 17.9	50.3 ± 18.1
150 kDa	2.11 ± 0.19	51.2 ± 5.5	24.3 ± 4.8
300 kDa	3.26 ± 0.18	43.6 ± 6.9	13.4 ± 2.9

**Table 4:** Results of determination of lipase activity in cut-off 15 and 300 kDa enzyme membranes for the cross-flow reactor.

Cut-Off	Amount of Lipase Immobilized [mg]	Productivity [µmol h^−1^]	Productivity/mg of Lipase [µmol h^−1^ mg^−1^]
15 kDa	0.84 ± 0.18	60.3 ± 9.5	71.9 ± 26.8
300 kDa	3.26 ± 0.18	99.7 ± 14.9	30.6 ± 6.3

## Data Availability

Not applicable.

## Supplementary Materials

The following are available online at https://www.mdpi.com/article/10.3390/ma15020671/s1, Figure S1: Synthesized modifier; Figure S2: ATR–FTIR spectra of pristine Al_2_O_3_ powder and Al_2_O_3_ powder modified with silane. Sections A and B are presented in Figure 1 in detail; Figure S3: Relation between roughness and contact angle of the investigated membranes.
