# Supplementary material for: Bioconjugation Strategy for Ceramic Membranes Decorated with Candida Antarctica Lipase B—Impact of Immobilization Process on Material Features
Source: Materials (Basel). 2022 Jan 17;15(2):671. doi: 10.3390/ma15020671 (PMC8779185; doi:10.3390/ma15020671)
Supplement: Supplementary file 1 [file materials-15-00671-s001.zip › materials-1502995-supplementary.pdf]

Supporting Information

Bioconjugation Strategy for Ceramic Membranes Decorated with *Candida Antarctica* Lipase B—Impact of Immobilization Process on Material Features

Joanna Kujawa [1], Marta Głodek [1], Izabela Koter [1], Guoqiang Li [1], Katarzyna Knozowska [1], Wojciech Kujawski [1,*]

[1] Nicolaus Copernicus University in Toruń, Faculty of Chemistry, 7 Gagarina Street, 87-100 Toruń, Poland;

* Correspondence: kujawski@chem.umk.pl; Tel.: +48-56-611-45-17 (W.K.)
Received: date; Accepted: date; Published: date

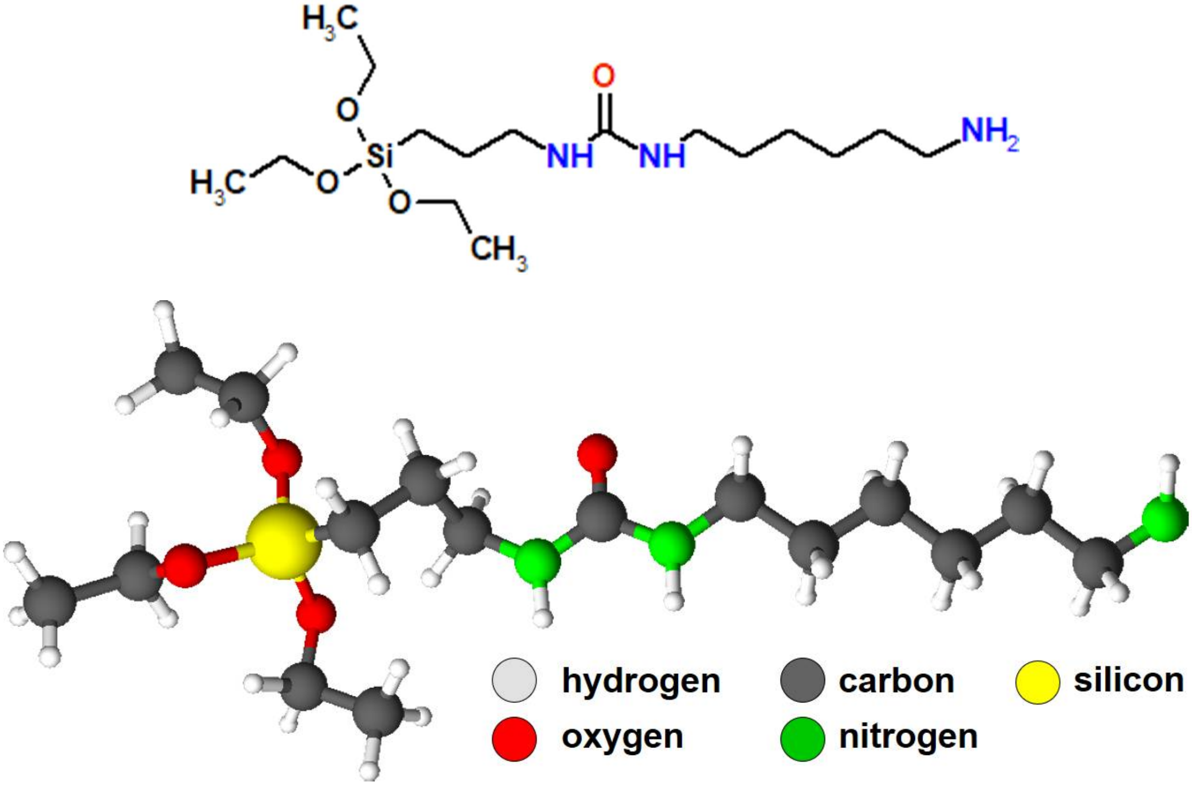

Figure S1. Synthesized modifier.

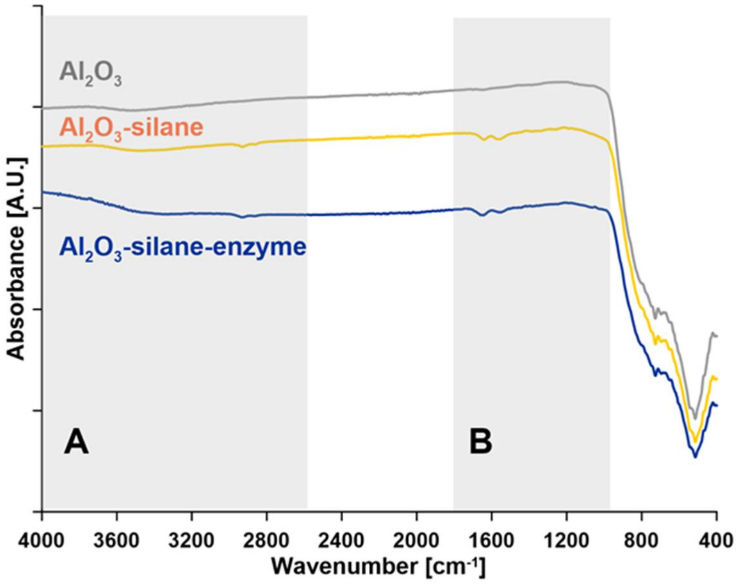

Figure S2. ATR–FTIR spectra of pristine Al_2O_3 powder and Al_2O_3 powder modified with silane. Sections A and B are presented in Figure 1 in detail.

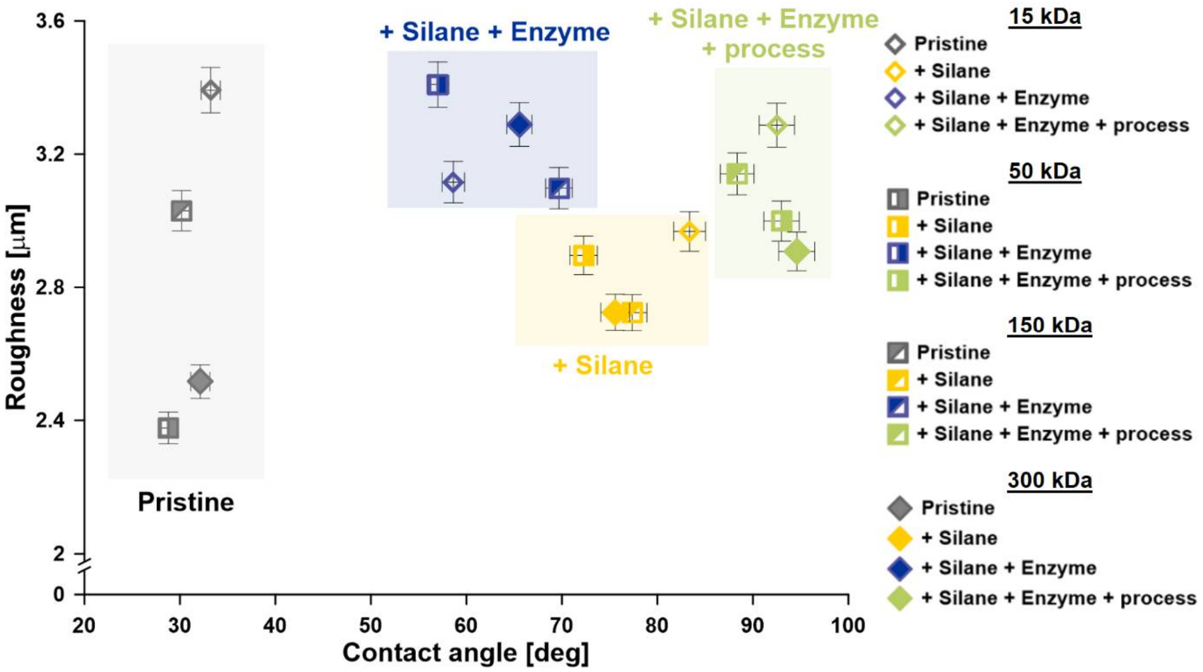

Figure S3. Relation between roughness and contact angle of the investigated membranes.
